# Highly Sensitive and Selective Dopamine Determination in Real Samples Using Au Nanoparticles Decorated Marimo-like Graphene Microbead-Based Electrochemical Sensors

**DOI:** 10.3390/s23052870

**Published:** 2023-03-06

**Authors:** Qichen Tian, Yuanbin She, Yangguang Zhu, Dan Dai, Mingjiao Shi, Wubo Chu, Tao Cai, Hsu-Sheng Tsai, He Li, Nan Jiang, Li Fu, Hongyan Xia, Cheng-Te Lin, Chen Ye

**Affiliations:** 1 College of Chemical Engineering, Zhejiang University of Technology, Hangzhou 310014, China; 2 Qianwan Institute, Ningbo Institute of Materials Technology and Engineering (NIMTE), Chinese Academy of Sciences, Ningbo 315201, China; 3 Center of Materials Science and Optoelectronics Engineering, University of Chinese Academy of Sciences, Beijing 100049, China; 4 Key Laboratory of Marine Materials and Related Technologies, Zhejiang Key Laboratory of Marine Materials and Protective Technologies, Ningbo Institute of Materials Technology and Engineering (NIMTE), Chinese Academy of Sciences, Ningbo 315201, China; 5 Laboratory for Space Environment and Physical Sciences, Harbin Institute of Technology, Harbin 150001, China; 6 School of Physics, Harbin Institute of Technology, Harbin 150001, China; 7 College of Materials and Environmental Engineering, Hangzhou Dianzi University, Hangzhou 310018, China; 8 State Key Laboratory for Mechanical Behavior of Materials, Xi’an Jiaotong University, Xi’an 710049, China

**Keywords:** dopamine determination, mesocarbon microbeads, gold nanoparticles, graphene nanowalls, electrochemical sensors

## Abstract

A sensitive and selective electrochemical dopamine (DA) sensor has been developed using gold nanoparticles decorated marimo-like graphene (Au NP/MG) as a modifier of the glassy carbon electrode (GCE). Marimo-like graphene (MG) was prepared by partial exfoliation on the mesocarbon microbeads (MCMB) through molten KOH intercalation. Characterization via transmission electron microscopy confirmed that the surface of MG is composed of multi-layer graphene nanowalls. The graphene nanowalls structure of MG provided abundant surface area and electroactive sites. Electrochemical properties of Au NP/MG/GCE electrode were investigated by cyclic voltammetry and differential pulse voltammetry techniques. The electrode exhibited high electrochemical activity towards DA oxidation. The oxidation peak current increased linearly in proportion to the DA concentration in a range from 0.02 to 10 μM with a detection limit of 0.016 μM. The detection selectivity was carried out with the presence of 20 μM uric acid in goat serum real samples. This study demonstrated a promising method to fabricate DA sensor-based on MCMB derivatives as electrochemical modifiers.

## 1. Introduction

Dopamine (DA) is distributed extensively in the central nervous system and peripheral tissues acting as a catecholamine neurotransmitter for message transfer, and itis involved in various important physiological functions including human metabolism, vasodilation, central nervous, motion control, as well as renal and hormonal systems [1,2,3]. DA is also a clinically important molecule in health care medicine, used in hypertension, bronchial asthma, cardiac surgery, and myocardial infarction [4]. The abnormal levels of DA in biological liquids and tissues are commonly related to several diseases and neurological disorders, like hypertension, schizophrenia, attention-deficit/hyperactivity, Parkinson’s disease, Alzheimer’s disease, and Huntington’s disease [5,6]. Due to the low concentration levels of DA in biological samples, usually in the range of several 10 s of nM, the development of reliable, accurate, and cost-effective sensing technology for the determination of DA with very high sensitivity and selectivity is essential in analytical and diagnostic applications [7,8]. Nowadays, in the field of DA detection, several analytical methods such as high-performance liquid chromatography, chemiluminescence, and capillary electrophoresis have been reported [9,10,11]. As the development of point of care testing (POCT) which is defined as medical diagnostic testing at/near the time and place of patient care [12,13], more requirements are raised for detection methods, including high sensitivity, low cost, ease-of-operation, and time efficiency, etc. Therefore, due to these competitive advantages, electrochemical analysis techniques have been regarded as a promising approach compatible with portable devices for the detection of electrochemically active DA molecules [14,15,16].

The most common electrode used for the recognition of electrochemically active compounds is the glassy carbon electrode (GCE) [7,17,18], which has a variety of advantages including inertness in a wide electrochemical window, chemical stability, and good electrical conductivity [19]. In order to further improve the sensing performance, several methods have been developed such as electrochemical activation [20], ultrasonication pretreatment [21], and surface modification with conductive nanomaterials [22,23,24]. Among them, the surface modification route has attracted a great deal of attention from academia and has become a rapidly growing research field, especially for biosensing applications [25,26,27,28]. In recent years, a variety of nanomaterials, such as nanoparticles (noble metals, transition metals, oxides, etc.) [29,30,31], nanotubes or nanofilaments (carbon nanotubes, noble metals, oxides, etc.) [32,33,34], and 2D materials (graphene, MXene, MoS_2_ nanosheets, etc.) [35,36,37], have been reported as nanomodifiers to boost the sensitivity of GCE towards DA detection. One of the most well-known 2D materials, graphene, which is composed of sp^2^-hybridized carbon atoms packed into a honeycomb lattice [38], has a strong interaction with adsorbed biomolecules and thus shows superior performance to improve the properties of DA sensors compared to other nanomaterials [7,39,40] because of its high specific surface area (up to 800 m^2^ g^–1^) [41], excellent electrical conductivity (≈2200 S cm^−1^) [42], and diverse defects/functional groups with enhanced electrocatalytic activity [43]. 

In general, graphene sheets and their derivatives including graphene oxide (GO) and reduced graphene oxide (rGO) used for electrochemical modifiers, can be massively and economically produced by exfoliation of graphite using the Hummers method, ball milling, and liquid phase sonication [44,45,46]. Yang et al. fabricated rGO-modified electrodes through drop-casting of GO dispersion on the GCE surface followed by electrochemical reduction, showing the sensing performance of DA with a linear range of 0.5–60 μM and a low detection limit of 0.5 μM [47]. Ping et al. developed a screen-printed graphene electrode using rGO ink chemically reduced by a mixture of hydrazine and ammonia solution for selective detection of DA, achieving a linear range of 0.5–2000 μM and a detection limit of 0.12 μM [48]. Noticeably, the sensitivity of graphene-modified electrodes reported above still need improvement for real sample analysis, since the basal level of DA concentration in human serum or plasma can be very low (in the range of 1 nM–1 μM) [18]. This limitation is because of the unavoidable formation of graphene agglomerates on the modified electrodes during the drying step when prepared by conventional drop casting, based on the strong π–π interactions between graphene sheets [49,50], thus leading to a significant reduction of the effective area between graphene and biomolecules, as well as the degradation of the sensitivity of DA sensors [51].

In this work, we synthesized a unique marimo-like structure consisting of graphene layers on the surface of commercial mesocarbon microbeads (MCMB) for the highly sensitive determination of DA molecules, through the proposed self-exfoliation process. MCMB is the spherical graphite particle synthesized from petroleum pitch with an average diameter of 10 s micrometers and high electrical conductivity, having been a commonly used material for lithium-ion batteries [52]. However, so far there is no report on the use of MCMB for applications in electrochemical sensors owing to the very low specific surface area (1.0–1.5 m^2^ g^–1^) [53]. Instead, in our study, graphene layers were partially exfoliated from the MCMB surface to form a marimo-like structure at 1000 °C with KOH for increasing the electroactive area, meanwhile eliminating the agglomeration behavior of drop-casted graphene electrodes. In addition, the decoration of the graphene surface with noble metal nanoparticles (NP) has been proven to be an efficient approach to improve the electrochemical sensitivity of DA sensors based on their excellent electroactivity and biocompatibility [54,55]. As a result, the GCE modified with marimo-like graphene (MG) microbeads for DA determination exhibits a wide dynamic range of 0.2–100 μM and a limit of detection (LOD) of 0.15 μM, and can be further enhanced to have a linear range of 0.02–10 μM and a LOD of 0.016 μM (16 nM) with the decoration of Au nanoparticles (Au NP). Moreover, the fabricated sensors exhibit good repeatability and specificity toward real sample analysis. To the best of our knowledge, this study is the first to report on sensing applications using MCMB derivatives as electrochemical modifiers.

## 2. Materials and Methods

### 2.1. Chemicals

Phosphate buffer solution (10× PBS, containing 137 mM NaCl, 102.7 mM KCl, 8.1 mM Na_2_HPO_4_, and 1.8 mM KH_2_PO_4_), H_2_SO_4_, KOH, NaCl, KCl, ascorbic acid (C_6_H_8_O_6_), uric acid (UA, C_5_H_4_N_4_O_3_), and glucose (C_6_H_12_O_6_) were purchased from Sinopharm Chemical Reagent Co., Ltd. (Shanghai, China). Dopamine (DA, 4-(2-aminoethyl)-pyrocatecho hydrochloride, C_8_H_12_ClNO_2_) was purchased from Shanghai Aladdin Biochemical Technology Co., Ltd. (Shanghai, China). HAuCl_4_·3H_2_O was purchased from SigmaAldrich Trading Co., Ltd. (Shanghai, China). All chemical reagents were analytical grade without further purification. Mesocarbon microbeads (MCMB) were purchased from Tianjin BTR New Energy Technology Co., Ltd. (Tianjin, China). Goat serum was purchased from Sangon Biotech Co., Ltd. (Shanghai, China). Milli-Q deionized water (DI water, ≈18.2 MΩ cm) was extensively utilized in our experiments. 

### 2.2. Fabrication of Au NP/Marimo-like Graphene Electrodes

Marimo-like graphene (MG) was made from MCMB powder mixed with KOH flakes (mass ratio 1:5) for 1 hat 1000 °C muffle furnace [56]. After treatment and removal of KOH, 60 mg MG was dispersed into 10 mL of DI water and then ultrasonically dispersed for 30 min. HAuCl_4_ and the ascorbic acid solution were prepared by adding 6 mg of HAuCl_4_ into 5 mL of DI water and 30 mg of ascorbic acid into 5 mL of DI water, respectively. 10 mL MG water dispersion was mixed with the HAuCl_4_ and ascorbic acid solution, followed by stirring for 30 min using a magnetic stirrer. After that, the solid product was filtered from dispersion and rinsed withDI water three times. The solid product is Au NP/MG [57]. The Au NP/MG was redispersed in DI water to obtain3 mgmL^−1^ dispersion for the next modification step.

GCE electrodes with a diameter of 3 mm (geometric area: 7.07 mm^2^) were applied as the substrate electrodes. Before modification, GCE electrodes were polished using 0.05 µm alumina slurry and cleaned in deionized water and ethanol by ultrasonication. Following that, GCE was activated via 100 times cyclic voltammetric scanning in 0.5 M H_2_SO_4_ with a potential range from −1.0 to 1.0 V vs. SCE and a scan rate of 100 mV s^−1^. 6 μL of Au NP/MG aqueous dispersion was drop-casted to the center of the GCE. After drying at 60 °C for 10 min, Au NP/MG/GCE was finally fabricated. MCMB/GCE, MG/GCE, and Au NP/MCMB/GCE electrodes were prepared as similar method.

### 2.3. Samples Preparation

We diluted 10× PBS 10 times using DI water to obtain1× PBS, which was adopted as a buffer solution. The 20 μM DA solution was prepared by adding0.038 g of DA into 100 mL of 1× PBS, and the DA solutions with lower concentrations were all obtained by diluting 20 μM DA solution using 1× PBS in the proper proportion. Gradually adding 1 M KOH solution or 0.5 M H_2_SO_4_ solution (1× PBS used as solvent) into 20 μM DA solution to adjust the pH and adding 1× PBS to adjust DA concentration as well; finally, the solutions with 10 μM DA with different pH from 3 to 11 were obtained for pH study. Real samples were prepared by spiking DA solution with different concentrations into 20 mL of goat serum. The pH of real samples is 7.

### 2.4. Dopamine Electrochemical Determination

The bare GCE, MCMB/GCE, MG/GCE, Au NP/MCMB/GCE, and Au NP/MG/GCE were applied as working electrodes. A saturated calomel electrode (SCE) and a Pt electrode were applied as reference and counter electrodes, respectively. CV and DPV tests were conducted to analyze the electrochemical behavior of different concentrations of DA on the modified GCE. CV curves (five cycles) were recorded from −0.2 to 0.4 V with a scan rate of 100 mV s^−1^, whereas DPV tests were conducted from −0.2 to 0.4 V with an increment step of 4 mV, amplitude of 50 mV, pulse period of 0.5 s, and pulse duration time is 0.05 s.

### 2.5. Characterizations

Field emission scanning electron microscope (FEI, Hillsboro, OR, USA) and high-resolution transmission electron microscope (JEOL, Tokyo, Japan)were applied to observe the modified material. Raman spectroscopy (Renishaw PLC, Wotton-under-Edge, UK) with a laser wavelength of 532 nm and X-ray photoelectron spectroscopy (XPS, Kratos Analytical, Manchester, UK) were used to characterize the chemical compositions and element chemical states. All the electrochemical experiments were carried out by a CHI660e electrochemical workstation (Shanghai Chenhua, Shanghai, China).

## 3. Results and Discussion

### 3.1. Characterization of MG and Au NP/MG

**Figure 1:**
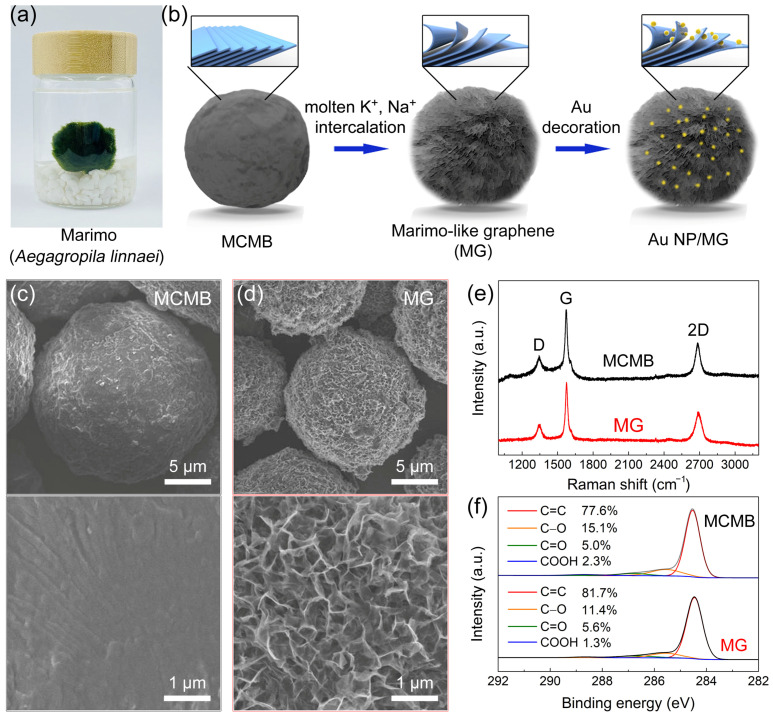
(**a**) Real photo of Marimo. (**b**) Schematic illustration of the fabrication of Marimo-like graphene (MG) and Au NP-decorated Marimo-like graphene (Au NP/MG). SEM images of (**c**) MCMB and (**d**) MG. (**e**) Raman spectra and (**f**) C1s XPS spectra of MCMB and MG.

Figure 1a,b show a schematic of the fabrication of marimo-like graphene (MG) and Au nanoparticles-decorated marimo-like graphene (Au NP/MG). The MCMB was mixed with five times the mass of the KOH solid. After raising the temperature to 1000 °C, the molten K^+^ ion intercalated into the graphite layer structure of MCMB, forming the partially exfoliated structure. Since the partially exfoliated MCMB is in a spherical shape with a fluffy graphene nanowalls shell, it was named marimo-like graphene (MG) in this work. Au nanoparticles-decorated marimo-like graphene (Au NP/MG) was acquired by decorating gold nanoparticles onto MG in a simple solution method through reducing chloraureate.

SEM images of MCMB and MG were shown in Figure 1c,d. The MCMB shows a relatively smooth spherical structure, whereas the MG is distinguishably rough. Under high resolution, the rough surface of MG is composed of graphene nanowalls, which were partially exfoliated by molten alkali intercalation [56]. The average diameter of MG is ≈15 μm, which is smaller than that of MCMB (≈20 μm). The volume decrease can be attributed to the carbon etching by molten alkali at high temperatures. The Raman spectra of MCMB and MG are shown in Figure 1e. The Raman peaks correspond to the characteristic D-band (≈1349 cm^–1^), G-band (≈1578 cm^–1^), and 2D-band (≈2698 cm^–1^) [58]. Moreover, the I_D_/I_G_ ratio is 0.27–0.32 and I_2D_/I_G_ ratio is 0.55–0.65, confirming that their main component is graphite. The high-resolution C1s XPS spectra of MCMB and MG are shown in Figure 1f. The curve results can be fitted into four deconvoluted components: sp^2^-hybridized bonds (C=C, at ≈284.4 eV), hydroxyl (C–O, at ≈286.1 eV), carbonyl (C=O, at ≈287.1 eV), and carboxylate group (COOH, at ≈288.7 eV) [27]. The ratio of oxygen-containing groups is 18–23%, and scarcely sp^3^-hybridized bond (C–C) was found, suggesting that the chemical composition of MCMB and MG is close to pristine graphite [59].

**Figure 2:**
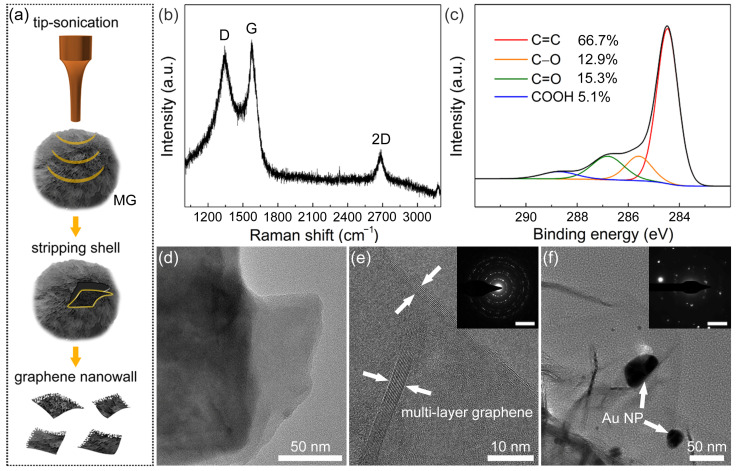
(**a**) Scheme of stripping and collecting graphene nanowalls shell from MG. (**b**) Raman spectrum and (**c**) C1s XPS spectrum of graphene nanowalls. (**d**) TEM image and (**e**) HRTEM image of MG (inset: SAED pattern; scale bar: 5 nm^–1^). (**f**) TEM image of Au NP/MG (inset: SAED pattern; scale bar: 5 nm^–1^).

However, compared to the particle size of MG, the thickness of the graphene nanowalls shell is limited. It should be noticed that the results of Raman and XPS spectra in Figure 1e,f reflect the information of the whole MG rather than the graphene nanowalls on the surface. Therefore, the graphene nanowalls shell was stripped from MG by tipsonication and collected for characterization (Figure 2a). As shown in the Raman spectrum of Figure 2b, a remarkable D-band can be observed, and the I_D_/I_G_ ratio is ≈ 0.96, indicating that a large number of defects exist in the graphene nanowalls shell [60]. These defects are mainly attributed to the exposed edge of graphene nanowalls. The C1s XPS spectrum in Figure 2c can be fitted as the same components as Figure 1f, and the ratio of oxygen-containing groups rose to ≈33%. The increase of oxygen-containing groups is the result of the inevitable oxidation reaction between MCMB and oxygenated compounds (e.g., OH^−^, O_2_, and H_2_O) during molten alkali intercalation. These oxygen-containing groups partially contribute to the defects in the graphene nanowalls shell as well.

Figure 2d displays the TEM bright field image of the top end of the graphene nanowalls shell. It can be observed that graphene nanowalls consist of a stacked laminated graphene structure. In the HRTEM image (Figure 2e), there are 10 lines of contrast at the edge of the graphene nanowalls shell, indicating that the layer number of multilayered graphene is 10 in this region. The interplanar spacing is approximately 0.34 nm, which corresponds to that of the graphite (002) facet [61]. The corresponding SAED pattern of graphene nanowalls is shown in Figure 2e. The presence of multiple plots revealed that graphene nanowalls are polycrystalline with various rotational stacking angles. In the TEM image of Au NP/MG (Figure 2f), several Au NPs decorate the graphene nanowalls, with a diameter range of 10–40 nm. Meanwhile, the corresponding SAED pattern shows that the Au NPs exist in polycrystalline form.

### 3.2. Au NP/MG/GCE Electrode Performance Optimization of DA Detection

**Figure 3:**
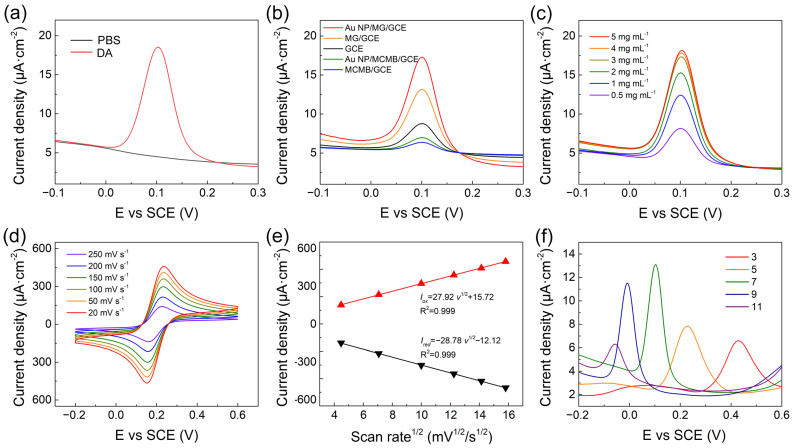
(**a**) DPV of Au NP/MG/GCE electrodes with and without 10 μM DA in PBS. (**b**) DPV curves of various modified electrodes with 10 μM DA in PBS. (**c**) DPV of Au NP/MG/GCE electrodes with various concentrations of MG to the same of AuNP with 10 μM DA in PBS. (**d**) Au NP/MG/GCE electrodes in 10 mM [Fe(CN)_6_]^3−/4−^ and 0.1 M KCl electrolyte solution at scan rates from 20 to 250 mV s^−1^. (**e**) Linear plots of *I_o__x_/I_r__ed_* versus scan rates (**f**) DPV of 10 μM DA on Au NP/MG/GCE electrodes with pH.

To explore the electrochemical performance of Au NP/MG/GCE towards DA, the DPV technique was conducted on the Au NP/MG/GCE electrodes in PBS with 10 μΜ DA. Compared to the curve from blank PBS, an oxidation peak at potential position 0.10 V appeared in the experimental group and specified as the characteristic peak for electrochemical analysis of DA, as shown in Figure 3a. As shown in Figure 3b, DPV curves of electrochemical behaviors at a potential interval of −0.1–0.3 V were performed in the presence of 10 μM DA on bare GCE, MCMB/GCE, Au NP/MCMB/GCE, MG/GCE, and Au NP/MG/GCE electrodes, respectively. The current intensities of MCMB/GCE and Au NP/MCMB/GCE electrodes were both lower than bare GCE, demonstrating the poor electrochemical activity and absorbability of MCMB towards DA. It is noticed that the current density is calculated from the geometric area of GCE. Compared to the MCMB/GCE electrodes, the current intensity of MG/GCE electrodes improved by ≈20%, indicating the much better electrochemical activity of MG than MCMB towards DA. It can be speculated that the specific surface area was greatly enhanced, and lots of edge defects as electrochemical active sites were exposed during the transition of MCMB to MG via molten alkali intercalation [56,62]. After hybridization with Au NPs, the electrochemical performance of Au NP/MG/GCE electrodes improved to the maximum, indicating that the electrodes with better electrical conductivity promoted the electron transfer of the DA oxidation reaction.

To further improve the electrochemical performance of the proposed sensor, experimental parameters including the preparation of modified electrodes, scan rate, and electrolyte pH were optimized. To confirm the suitable mass of MG in the fabrication of Au NP/MG/GCE electrodes, various concentrations of MG varying from 0.5–5 mg mL^–1^ at 0.5 μL volume were drop-casted on GCE and dried to perform DPV response in PBS with 10 μM DA. As shown in Figure 3c, the MG concentration was selected as 3 mg mL^–1^. The electrochemical behavior of various electrodes was performed by CV in 10 mM [Fe(CN)_6_]^3−/4−^ containing 0.1 M KCl electrolyte solution at scan rates ranging from 20 to 200 mVs^–1^ (Figure 3d). The peak currents density of *I_o__x_* and *I_r__ed_* both increased linearly with the square root of scan rates (Figure 3e), demonstrating that the redox reaction on the Au NP/MG/GCE electrodes was controlled by diffusion. The effect of pH on the electrochemical response of Au NP/MG/GCE electrodes was performed in the range from 3 to 11 with an increase factor of 2, as shown in Figure 3f. The peak potential position shifted negatively with the increased electrolyte pH, due to an improvement in the deprotonation reaction of DA [63]. The maximum value of peak current is at pH 7 and was chosen as the optimal pH. A possible mechanism of the maximum peak at pH 7 is the combination of the ion concentration effect and electrode surface adsorption process. When pH < 7, excess protons (H^+^) in the solution will inhibit the deprotonation reaction of DA. When pH > 7, DA molecules are negatively charged, and the electrode surface is also negatively charged due to the oxygen-containing group of MG. Therefore, although the deprotonation reaction of DA is promoted, the repelling effect of charge interaction makes it difficult for DA adsorption on the electrode, resulting in the decrease of electrochemical response current.

### 3.3. Electrochemical Determination of DA with Different Concentrations

**Figure 4:**
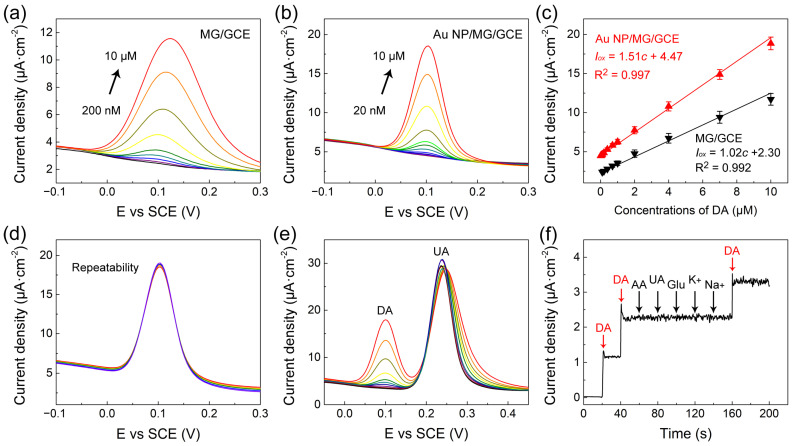
(**a**,**b**) DPV curves of MG/GCE and Au NP/MG/GCE electrodes with various concentrations of DA, respectively. (**c**) The corresponding peak current versus DA concentration. (**d**–**f**) The repeatability and good anti-interference of Au NP/MG/GCE electrodes.

The quantitative electrochemical determination of DA on the MG/GCE and Au NP/MG/GCE electrodes was performed by DPV measurements, respectively, as presented in Figure 4a,b. The peak current value on the MG/GCE and Au NP/MG/GCE electrodes both enhanced with the increasing concentration of DA. As indicated in Figure 4c, the calibration curve of DA on the MG/GCE and Au NP/MG/GCE electrodes was concluded from the average of peak current value, and the linear range of DA determination was in a range of 0.2–10 μM and 0.02–10 μM. The linear regression equation on the MG/GCE electrodes was *I_o__x_*(μA) = 1.02*c* (μM) + 2.30 (R^2^ = 0.992), and the linear regression equation on the Au NP/MG/GCE electrodes was *I_o__x_*(μA) = 1.51*c* (μM) + 4.47 (R^2^ = 0.997). The limit of detection (LOD) of DA on the MG/GCE and Au NP/MG/GCE electrodes was determined as 0.15 μM and 0.016 μM, respectively. Specifically, the DA determination experiments were performed by the DPV technique on six individual electrodes. Compared with other DA electrochemical sensors focusing on graphene-based modified electrodes, as listed in Table 1, our Au NP/MG/GCE electrodes achieved a relatively low detection LOD of DA with efficiency.

### 3.4. Repeatability, Anti-Interference, Recovery, and Real Sample Analysis

To evaluate the repeatability of Au NP/MG/GCE electrodes for DA determination (10 μM), DPV curves at a potential interval of −0.1–0.3 V were repeatedly measured 10 times on the same electrodes. As presented in Figure 4d, the oxidation peak potential of DPV curves was consistent at 0.10 V, and the curves overlapped well. The relative standard deviation (RSD) of peak currents was ≈2.6%, indicating good repeatability of Au NP/MG/GCE electrodes. The anti-interference of Au/MG/GCE electrodes in the presence of 20 μM UA as interfering substances was experimented with by DPV curves in goat serum real samples with spiked DA in the range from 0.1 to 10 μM, as indicated in Figure 4e. Compared to the values of the calibration curve in Figure 4c, the anti-interference results indicated that the presence of UA did not intervene in DA determination. Furthermore, the anti-interference of Au NP/MG/GCE electrodes in the presence of other potential interfering substances such as 1 µM ascorbic acid (AA), 1 µM glucose (Glu), 1 μM UA, 1 µM K^+^, and 1 µM Na^+^ in 1× PBS containing 0.1 µM DA was conducted by chronoamperometry measurements, as presented in Figure 4f. The results demonstrated that our constructed sensors have good anti-interference against other molecules during electrochemical determination. The standard addition method was applied to verify the recovery property of Au NP/MG/GCE electrodes.DPV curves of spiked serum samples with different DA concentrations (0.12–7.6 μM) were recorded under optimal conditions. As shown in Table 2, the prepared DA sensor exhibited good recoveries (99.7–106.5%) and low RSD values (0.82–1.54%), showing great potential for practical applications.

## 4. Conclusions

In this work, a DA sensor based on an Au NP/MG/GCE electrode was proposed. MG was prepared through partial exfoliation commercial MCMB via molten alkali intercalation. The exfoliated graphene nanowalls structure on MG microbeads not only increases specific surface area but also provides numerous electrochemical active sites. Compared to MCMB-based electrodes, MG-based electrodes display high sensitivity toward the oxidation of DA molecules. With the assistance of Au NP, the Au NP/MG/GCE electrode exhibited optimized properties for DA determination with a wide linear range from 0.02 to 10 μM and an ultralow detection limit of 0.016 μM. The good recovery (99.7–106.5%) and practicability of Au NP/MG/GCE electrode for DA detection in a real sample have been validated in goat serum samples. Moreover, the anti-interference of Au NP/MG/GCE electrode was further investigated by spiking other biological molecules and ions. We believe this study can provide a promising pathway to construct an electrochemical sensor based on MCMB derivatives.

## Figures and Tables

**Table 1:** Comparison of linear range and detection limit with other graphene-based DA electrochemical sensors.

Modifiers	Decoration	Substrate	Measurements	Linear Range (μM)	LOD (μM)	Ref
Graphene sheets		GCE	Amperometry	5.0–710	2.0	[35]
rGO		GCE	DPV	0.5–60	0.50	[47]
rGO		screen-printed electrode	DPV	0.5–2000	0.12	[48]
rGO nanoribbons		screen-printed electrode	DPV	0.5–300	0.15	[64]
rGO	Au NPs	GCE	DPV	6.8–41	1.4	[65]
rGO	Ag NPs	GCE	DPV	10–70	1.0	[29]
rGO	Pt NPs	GCE	DPV	0.03–8.13	0.03	[66]
rGO	Pd–Pt NPs	GCE	DPV	4–200	0.04	[67]
rGO	Au–Pt nanoclusters	GCE	DPV	0.07–49,800	0.02	[68]
Marimo-like graphene	-	GCE	DPV	0.2–100	0.15	This work
Marimo-like graphene	Au NPs	GCE	DPV	0.02–10	0.016	This work

**Table 2:** Recovery results of DA in real samples by using Au NP/MG/GCE electrodes.

Samples	Added (μM)	Founded (μM)	RSD (%)	Recovery (%)
Goat serum	0.120	0.123	1.06	102.5
	0.740	0.784	1.18	106.0
	1.30	1.38	1.54	106.2
	3.70	3.94	1.40	106.5
	7.60	7.58	0.82	99.7

## Data Availability

Data sharing not applicable.
